# Does the Smoke Ever Really Clear? Thirdhand Smoke Exposure Raises New Concerns

**DOI:** 10.1289/ehp.119-a70

**Published:** 2011-02

**Authors:** Adrian Burton

**Affiliations:** **Adrian Burton** is a biologist living in Spain who also writes regularly for *The Lancet Oncology*, *The Lancet Neurology*, and *Frontiers in Ecology and the Environment*

You may never have heard of thirdhand smoke, or THS, but chances are you’ve smelled it. THS is, in the words of *The New York Times*, “the invisible yet toxic brew of gases and particles clinging to smokers’ hair and clothing, not to mention cushions and carpeting, that lingers long after secondhand smoke [SHS] has cleared from a room.”^1^ Recent research exploring potential dangers of THS has received a flurry of coverage in the international media^2^,^3^,^4^ and the scientific press.^5^,^6^,^7^ And in the United States, court cases are beginning to appear in which plaintiffs are citing these alleged dangers,^8^,^9^ despite a lack of human health studies on the long-term health effects of THS exposure.

So how dangerous might THS really be? The answer, still to be pronounced, will depend on many factors.

## A Brief History of THS

THS was a topic of interest long before it received its present name. The seed of the idea that cigarette smoke toxicants might linger on room and car surfaces long after the smoke itself was gone was planted in 1953, when it was reported that smoke condensate painted onto mice caused cancer.^10^

In 1991 the house dust of smokers’ homes was first found to be contaminated with nicotine.^11^ Later, in 2004, nicotine was quantified in the dust of nonsmokers’ homes and homes in which mothers smoked in the house over the preceding 3 months.^12^ In homes with the highest SHS exposure, in which the mothers smoked in areas where their children were present, nicotine in dust averaged 64.0 μg/m^2^ in living rooms and 15.8 μg/m^2^ in infants’ bedrooms. Surfaces in living rooms and infants’ bedrooms averaged nicotine coatings of 73.05 μg/m^2^ and 56.26 μg/m^2^, respectively. The same study showed the dust and surfaces of homes in which smokers had tried to limit their children’s exposure (for instance, by sometimes smoking outdoors) were also contaminated, although to a lesser degree. However, no nicotine was found in the dust or on the surfaces of homes never exposed to tobacco smoke.^12^

In 2008 similar findings were reported for cars.^13^ Nicotine was detected in significantly greater quantities in the dust (mean 19.51 μg/g) and on the dashboards (mean 8.61 μg/m^2^) of 78 vehicles belonging to people who smoked in their vehicles than in the dust (mean 3.37 μg/g) and on the dashboards (mean 0.06 μg/m^2^) of 20 vehicles of nonsmokers. Eight smokers had imposed a smoking ban in their vehicles for at least 12 months. Their vehicles nevertheless were contaminated with nicotine (mean 11.61 μg/g in dust and 5.09 μg/m^2^ on the dashboard). The authors point out, however, that the cars may have been contaminated by smoke that entered the car from outside and that smoking bans may not have been complied with 100% of the time.

A 2010 study showed THS also remains after smokers move out of their homes, even after being vacant for two months and being prepared for new residents, sometimes with new carpeting and paint.^14^ Meanwhile, other lines of research have confirmed some smoke compounds adsorb onto surfaces and then desorb back into the air over time, providing a source of tobacco toxicants that lingers long after people finish smoking.^15^,^16^

The term *thirdhand smoke* may have first appeared in print in 2006,^17^ but it became more widely known in 2009 when it was used by Jonathan Winickoff, an associate professor of pediatrics at Harvard Medical School, and colleagues in a paper published in *Pediatrics*.^18^ In that work, the researchers reported that 65.2% of nonsmokers and 43.3% of smokers believed THS could harm children and that such beliefs were independently associated with the imposition of home smoking bans. The authors also wrote that emphasizing the potential dangers of THS to children’s health might be important in encouraging parents not to smoke around their children.

A new development emerged when Mohamad Sleiman, a chemist with the Indoor Environment Department of the Lawrence Berkeley National Laboratory (LBNL) Environmental Energy Technologies Division, and colleagues reported that nicotine adsorbed onto surfaces reacted with nitrous acid—an air pollutant found in vehicle exhaust and produced by improperly vented gas stoves and burning tobacco—to form tobacco-specific nitrosamines (TSNAs) including 1-(*N*-methyl-*N*-nitrosamino)-1-(3-pyridinyl)-4-butanal (NNA), 4-(*N*-nitrosomethylamino)-1-(3-pyridinyl)-1-butanone (NNK), and *N*-nitrosonornicotine (NNN).^19^ There is some evidence NNA is mutagenic.^20^ NNK and NNN are classified by the International Agency for Research on Cancer as human carcinogens^21^ and by the National Toxicology Program as reasonably anticipated to be human carcinogens.^22^

Later in 2010 Sleiman et al. reported that ozone, another indoor air pollutant, reacted with some 50 compounds in SHS to produce ultrafine particles smaller than 100 nm, the compositions of which are yet to be determined.^23^ The effects of ultrafine particles are thought to vary depending on their composition and characteristics, but their tiny size likely facilitates their uptake and distribution throughout the body to potentially sensitive target sites including the bone marrow, lymph nodes, spleen, heart, and central nervous system.^24^

Sleiman et al. also speculated these ultrafine particles may be capable of depositing on surfaces and later resuspending into the air.^23^ In the same year, another research team provided the first preliminary quantitative data showing these particles did just that, although reaching airborne concentrations 100 times lower than levels in SHS.^25^

By the latter part of 2010, with *thirdhand smoke* an established moniker, researchers began to define the phenomenon with a “three Rs” description: “Thirdhand smoke consists of residual tobacco smoke pollutants that **r**emain on surfaces and in dust after tobacco has been smoked, are **r**e-emitted back into the gas phase, or **r**eact with oxidants and other compounds in the environment to yield secondary pollutants,” Sleiman says.

## Working It Out

Although concern that THS might be a hazard has grown, proof of harm remains to be formally demonstrated. The papers by Sleiman et al.^19^,^23^ focused on chemistry; they did not study health implications. However, figures reported in their nitrous acid/TSNA paper^19^ allow a back-of-the-envelope calculation that provides a starting point for debate on the potential of THS to cause harm.

In this work, Sleiman and his colleagues sampled the interior of an old pickup truck whose owner typically smoked more than 10 cigarettes a day inside the vehicle. They placed a filter-paper patch on the dashboard; three days later, with the owner having smoked as usual, they removed the filter paper and also took a wipe sample of the stainless steel glove compartment door. Both the filter-paper and wipe samples were analyzed, showing that ambient nitrous acid levels were able to produce TSNAs by reaction with nicotine. No NNN was detected in this experiment, but the filter paper returned values of around 1 ng/cm^−2^ for NNK and 5 ng/cm^−2^ for NNA. The glove compartment door returned about 0.2 ng/cm^−2^ for NNK and 1.0 ng/cm^−2^ for NNA.

Considering the filter-paper results for the truck and factoring in many assumptions, a calculation for potential exposure emerges (see Box 1). At this point, estimating the cancer risk of such an exposure would be speculative—no cancer potency factor (CPF) is available for NNA,^19^ and the CPF for NNK refers to a combination of lung, pancreas, liver, and nasal cancers in association with oral exposure over a lifetime of 70 years.^26^ Sleiman and colleagues caution, moreover, that an important limitation of the calculation in Box 1 is the assumption that 100% of NNK and NNA on the surface of the hand is absorbed into the body and/or ingested.

Box 1 **An Estimate of Exposure**

**NNA absorbed on filter paper = 5 ng/cm**^−**2**^

**Area of passenger’s hand = 160 cm^2^**

**One firm handplant on the dashboard could conceivably pick up 5 × 160 = 800 ng NNA,** assuming all the NNA on the dashboard is picked up by the hand

**Assume the hand is wiped across a 1-m swath of the dash.** With an average hand width of 10 cm, this equals 10 handplants made on surfaces similar to the dashboard. A passenger could pick up 10 × 800 = 8,000 ng (or 8 μg) NNA.

**For NNK, divide this figure by 5** (only 1 ng/cm^−2^ NNK was found on the dashboard): 8/5 = 1.6 μg

**Assuming the passenger weighs 80 kg, the potential doses received are:**

**NNA:** 8 μg over 80 kg body weight = 0.1 μg/kg for 3 days, or 0.033 μg/kg/day

**NNK:** 1.6 μg over 80 kg body weight = 0.02 μg/kg for 3 days, or 0.0066 μg/kg/day

But although the predicted figures clearly could be lower, Sleiman says at least some of the input figures seem reasonable. He points out, “The quantities of TSNAs on the paper were only those collected after three days of one person smoking ten cigarettes per day. How much more might be accumulated after months of smoking, perhaps by more than one smoker smoking more than ten per day?”

“Different materials absorb different amounts of nicotine [therefore producing different amounts of TSNAs],” adds coauthor Hugo Destaillats, also of the LBNL. “We only examined paper and stainless steel; other materials in cars and homes absorb other quantities.” For instance, wool, cotton, silk, linen, acetate, and polyester all absorb SHS compounds,^27^,^28^ and nicotine is reported to be adsorbed by carpet and wallboard in quantities 2–3 orders of magnitude greater than the amount that would have been adsorbed by the pickup truck’s stainless steel glove compartment door.^29^

Further, although nitrous acid levels typically reach 5–15 ppb by volume indoors and 30 ppb by volume in vehicles, concentrations as high as 100 ppb by volume have been measured indoors.^30^ Moreover, nitrous acid photodecomposes during the day, so concentrations could be especially high at night in polluted cities, speculates coauthor Lara Gundel, also an LBNL researcher; TSNA production could increase with higher nitrous acid concentrations.

Gundel adds that SHS contains many more toxic and carcinogenic compounds—such as benzo[*a*]pyrene, 1,3-butadiene, benzene, formaldehyde, cadmium, arsenic, and lead—that the researchers did not consider in their studies. “Alongside NNK and other TSNAs, they could increase the dangers of thirdhand smoke residue,” Gundel says. Moreover, she says, the dermal CPF for at least one compound in SHS—benzo[*a*]pyrene—is actually about 15-fold higher than its oral counterpart.^31^

Winickoff is concerned that small children might be particularly exposed and more susceptible to toxicants in THS. “Infants crawl over, touch, and mouth contaminated surfaces and are known to consume up to a quarter gram per day of dust—twice as much as do adults,” he says. “They could therefore be getting much higher doses of thirdhand smoke toxicants than older children and adults.” Gundel also suggests that cleaning staff working in hotels where smoking is allowed could receive high THS exposures, for example by handling THS-contaminated bedding.

## Overshooting?

Clearly, not all the worst-case scenario inputs used in Box 1 may apply. Michael Siegel, a professor of community health sciences at Boston University School of Public Health, says there is no evidence to support the assumption that 100% of the NNK on the surface of the hand would be absorbed into the body and/or ingested. He further argues, “The most likely source of meaningful human exposure—ingestion—would only be a major issue for infants, and the time period during which high levels of ingestion of chemicals on the hands occurs is only about one year” (although Gundel points out a smoker’s spouse might certainly be exposed for 50 years over the span of a marriage).

A more important consideration, suggests Siegel, is whether the potential threat posed by THS adds significantly to the hazards of smoking and SHS exposure. Smokers who are exposed to THS on surfaces after smoking has ceased would already have been exposed to many times the quantities of the same chemicals through the act of smoking itself, he explains. Likewise, nonsmokers who are exposed to SHS—including the children of smokers—also would take in far greater quantities of NNK and other toxics via smoke inhalation than through THS. “This would make any small additional NNK exposure meaningless,” Siegel says.

Siegel believes one issue that *is* potentially meaningful is whether significant exposure to toxic THS constituents could occur as a result of smoke absorbed by a smoker’s clothing. “This question is important because it determines whether or not smokers who smoke only outside the home nevertheless place their children at potential risk,” he says. “The research that is needed is a study to determine the level of infant carcinogen exposure resulting in the setting of parents who only smoke outside the home.”

Finding subjects for such research will not be too hard. Data from the National Health and Nutrition Examination Survey 2007–2008 indicate more than half of U.S. children aged 3–19 years, some 32 million children, are exposed to SHS.^32^ Globally, an estimated 40% of children, 35% of nonsmoking women, and 33% of nonsmoking men are regularly exposed to SHS.^33^

## Cutting through the Smoke

Of the Sleiman et al. paper,^19^ Catherine Armstrong, a spokeswoman for British American Tobacco, says, “[This work] did not study any health outcomes. As the authors themselves note, more research is needed before conclusions on possible health hazards can be drawn.” That research is about to start. The California Tobacco-Related Disease Research Program, which is funded by the California tobacco tax, recently made US$3.75 million of funding available for studying THS and cigarette butt waste.^34^

Georg Matt, a professor of psychology at San Diego State University, points out that even in the absence of any hard evidence of actual long-term health effects of THS, many nonsmokers—and former smokers—have already been sensitized to the phenomenon. “We ask for nonsmoker hotel rooms, nonsmoker apartments, and we prefer nonsmoker cars when we buy a used car. Hotels and car rental companies know that cleaning up [smokers’] cars and rooms is very expensive, and real-estate agents know that smoking affects property values.”

Regardless of whether THS is conclusively shown to cause illnesses, it is already changing attitudes, behaviors, norms, expectations, purchasing behavior, and the economic value of personal property and real estate, Matt says. In combination, these are powerful factors that have the potential to reduce tobacco use and lower the health risks associated with smoking itself as well as SHS and THS exposure.

“The most important impact of the efforts to prevent exposure to thirdhand smoke,” Matt says, “may be . . . the reduction of health risks from active smoking and secondhand smoke exposure.” For these forms of tobacco smoke exposure, at least, the discussion about whether they may be dangerous is well and truly concluded.

## Figures and Tables

**Figure:**
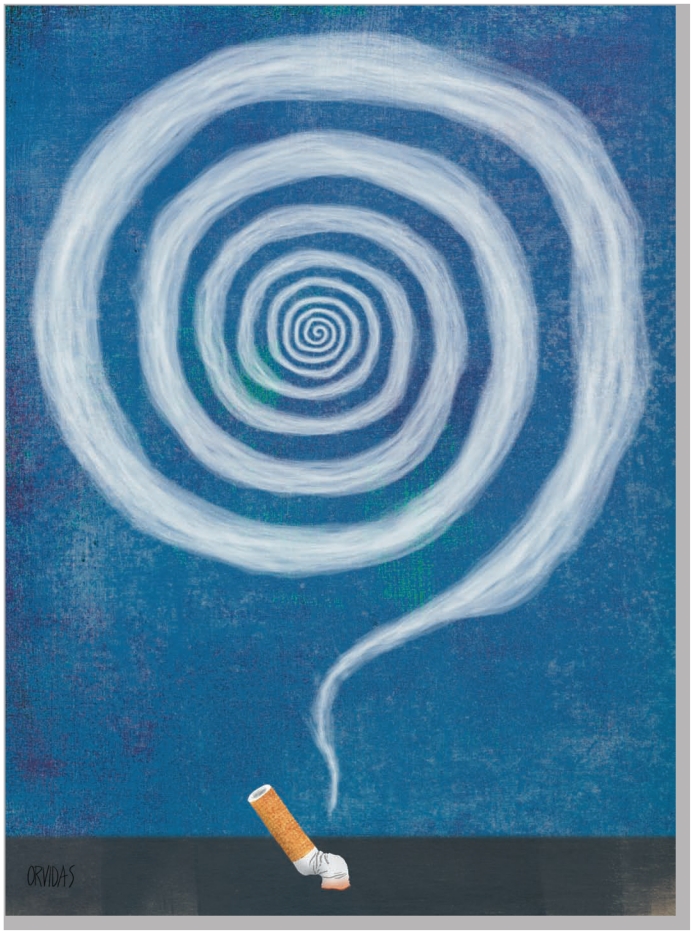
Thirdhand smoke consists of residual tobacco smoke pollutants that 1) remain on surfaces and in dust after tobacco has been smoked, 2) are re-emitted back into the gas phase, or 3) react with oxidants and other compounds in the environment to yield secondary pollutants.

**Figure:**
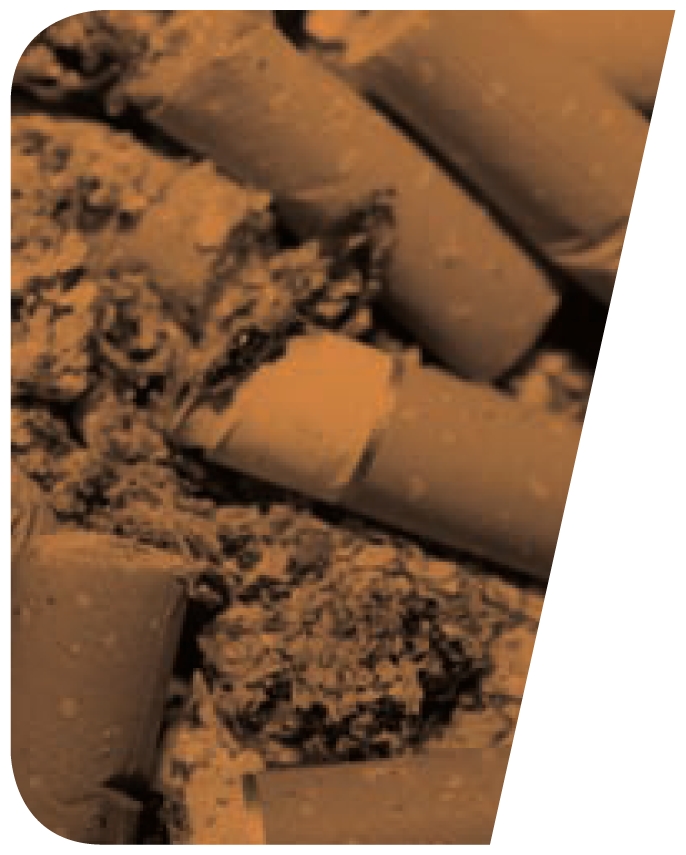
Michael Siegel of Boston University School of Public Health believes one potentially meaningful question is whether significant exposure to toxic THS constituents could occur as a result of smoke absorbed onto a smoker’s clothing. “This question is important because it determines whether or not smokers who smoke only outside the home nevertheless place their children at potential risk,” he says.

**Figure:**
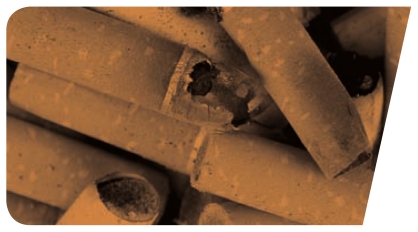
Georg Matt of San Diego State University points out that in the absence of any hard evidence of actual long-term health effects of THS, many nonsmokers—and former smokers—have already been sensitized to the phenomenon. “The most important impact of the efforts to prevent exposure to thirdhand smoke may be . . . the reduction of health risks from active smoking and secondhand smoke exposure,” he says.
